# RNA-sequencing analysis reveals the long noncoding RNA profile in the mouse myopic retina

**DOI:** 10.3389/fgene.2022.1014031

**Published:** 2022-10-13

**Authors:** Yuanjun Li, Ying Lu, Kaixuan Du, Yewei Yin, Tu Hu, Qiuman Fu, Yanni Zhang, Dan Wen, Xiaoying Wu, Xiaobo Xia

**Affiliations:** ^1^ Eye Center of Xiangya Hospital, Central South University, Changsha, China; ^2^ Hunan Key Laboratory of Ophthalmology, Xiangya Hospital, Central South University, Changsha, China; ^3^ National Clinical Research Center for Geriatric Disorders, Xiangya Hospital, Central South University, Changsha, China

**Keywords:** Long noncoding RNA, messenger RNA, RNA sequencing, myopia, retina

## Abstract

**Aim:** Myopia is a prevalent public health problem. The long noncoding RNA (lncRNA) mechanisms for dysregulated retinal signaling in the myopic eye have remained elusive. The aim of this study was to analyze the expression profiles and possible pathogenic roles of lncRNAs in mouse form-deprived myopia (FDM) retinas.

**Methods:** A mouse FDM model was induced and retinas from the FDM right eyes and the contralateral eyes were collected for RNA sequencing. Gene Ontology (GO), Kyoto Encyclopedia of Genes and Genomes (KEGG) pathway enrichment, and lncRNA-mRNA coexpression network analyses were conducted to explore the biological functions of the differentially expressed lncRNAs. In addition, the levels of differentially expressed lncRNAs in the myopic retinas were validated by quantitative real-time PCR (qRT–PCR). Fluorescence *in situ* hybridization (FISH) was used to detect the localization of lncRNAs in mouse retinas.

**Results:** FDM eyes exhibited reduced refraction and increased ocular axial length compared to control fellow eyes. RNA sequencing revealed that there were 655 differentially expressed lncRNAs between the FDM and control retinas. Functional enrichment analysis indicated that the differentially expressed RNAs were mostly enriched in cellular processes, cytokine-cytokine receptor interactions, retinol metabolism, and rhythmic processes. Differentially expressed lncRNAs were validated by qRT–PCR. Additionally, RNA FISH showed that XR_384718.4 (Gm35369) localized in the ganglion cell (GCL) and inner nuclear layers (INL).

**Conclusion:** This study identified the differential expression profiles of lncRNAs in myopic mouse retinas. Our results provide scientific evidence for investigations of myopia and the development of putative interventions in the future.

## Introduction

Myopia is the most prevalent refractive error and a leading cause of visual impairment worldwide (Dolgin, 2015). In recent decades, there has been a pandemic increase in myopia prevalence, and uncorrected refractive error has become a major public health concern, affecting a large proportion of the world population (Lou et al., 2016; Cheng et al., 2020). Myopia is characterized by excessive elongation in ocular axial length (AL) accompanied by scleral thinning and stretching of other ocular tissues. High myopia (−6.00 D or worse) can lead to severe visual impairments caused by complications such as posterior staphyloma, glaucoma, choroidal neovascularization, myopic retinal degeneration, and detachment (Grossniklaus and Green, 1992; Wu et al., 2000). Multiple factors, including genetic anomalies, intensive near work, insufficient outdoor activities, etc., are involved in the development of myopia (Zhao et al., 2020). Although previous studies have implicated dopamine (Huang et al., 2020), nitric oxide (NO) (Carr and Stell, 2016), retinoic acid (RA) (Wang et al., 2014), glutamate (Guoping et al., 2017), the extracellular matrix (Liu et al., 2017), and, recently, scleral hypoxia (Wu et al., 2018) in the etiology of myopia, the mechanisms and pathogenesis of myopia still require further investigation.

Long noncoding RNAs (lncRNAs) are a class of transcripts greater than 200 nt in length that have little or no protein-coding potential (Carninci et al., 2005; Guttman et al., 2013). LncRNAs have been found to play important roles in a variety of biological processes, including chromatin organization, transcriptional/translational regulation, stem cell maintenance, differentiation, and cell fate reprogramming (Brockdorff et al., 1991; Geisler and Coller, 2013; Flynn and Chang, 2014).

Previous studies have shown that lncRNAs are associated with diverse ocular diseases, including diabetic retinopathy, retinal neovascularization, glaucoma (Zheng M. et al., 2020), cataracts (Tu et al., 2020), proliferative vitreoretinopathy (Ni et al., 2021) and retinoblastoma (Wang H. et al., 2021). RNA sequencing (RNA-seq) of a guinea pig form-deprived myopia (FDM) model and a lens-induced myopia (LIM) model has also suggested that there is differential lncRNA expression in the ocular posterior pole (Geng et al., 2020). lncRNA-associated extracellular matrix (ECM), ECM-receptor interaction, kinase activity, metabolism and multiple functional pathways are involved in myopia pathogenesis (Geng et al., 2020). lncRNAs can affect gene expression by functioning as competitive endogenous RNAs (ceRNAs) with microRNAs (miRNAs), competing with mRNAs for miRNA binding (Cesana et al., 2011). Since miRNA profiling in LIM mice, LIM guinea pigs, and highly myopic patients has suggested the existence of differentially regulated miRNA patterns (Tanaka et al., 2019; Guo et al., 2020; Zhu et al., 2020), lncRNAs might modulate gene expression through RNA interactions and thus regulate myopia.

The retina is a thin layer of complex neural tissue that receives light-stimuli and processes visual signal, and transmitted signal to the sclera. Numerous studies have suggested the retina playing important roles in the pathology in myopia, such as circuiting electrical and chemical synapses (Zhi et al., 2021). Moreover, lncRNAs play pathogenic roles in several retinal diseases, such as the lncRNA XIST and nuclear paraspeckle assembly transcript 1, which play roles in diabetic retinopathy (Li, 2018; Dong et al., 2020). Nevertheless, the detailed expression profiles and pathogenic mechanisms of lncRNAs in myopic retina remain largely elusive. As there were several sequencing studies focusing on the changes in sclera of myopia, while the role of retinal structure in the pathology of myopia remains unclear and complex, the sequencing analysis here hope to lay a foundation for the future study about the mechanisms (especially for the non-coding RNAs) in myopic retina.

Previous myopia studies have built well-established experimental myopic animal model, including FDM and lens-induced myopia (LIM). The two models differ from each other in the methods and behind mechanisms: FDM is induced by deprivation of form vision, while LIM by wearing concave lens to form image behind the retina and to induce excessive accommodation and extension of axial length (Xiao et al., 2014). Study with chicks indicated that the dopaminergic mechanisms mediating the protective effects of brief periods of unrestricted vision might differ for FDM vs. LIM, implying that the two might be different in the growth control mechanisms (Nickla and Totonelly, 2011). Form-deprivation has been well-developed and effective to induce myopia, and extensively used in research into the mechanisms, pathology, sequencing analysis of myopia, thus the current study applied FDM model to explore the lncRNA and mRNA expression pattern in myopic retina. Early study of experimental myopia has tested two mouse strains, C57BL/6 and DBA/2, and concluded that DBA/2J were unaffected by occlusion for 7 or 14 days; prolonged occlusion produces a significant myopic shift in C57BL/6 mice, but not in DBA/2J (Schaeffel et al., 2004). Thus, recent myopic mice studies applied the C57BL/6 strains in the model building. Our process of building the FDM model was almost the same to the procedure of Wu’s report with the male C57BL/6, which was started at the age of 3 weeks postnatal, and deprived for 4 weeks by wearing monocular occlusion in the right eyes (Wu et al., 2015).

In the present study, a mouse FDM model was established and characterized. RNA sequencing was applied to compare lncRNA and mRNA expression patterns in retinal tissue between FDM and control mice. Next, the differentially expressed lncRNAs and mRNAs were utilized to conduct pathway enrichment and coexpression network analyses by bioinformatics methods. Furthermore, lncRNA expression was validated by qRT–PCR and localized by RNA FISH. This study aimed to provide experimental evidence of lncRNA profiles in the retina in the context of myopia, which might enable further investigation and the development of a therapy for this ocular disease.

## Materials and methods

### Animals

Male C57BL/6J mice (3 weeks of age, weight 10–15 g) from the Animal Unit of Central South University were used in this study. Mice were treated under the rules of the Association for Research in Vision and Ophthalmology Statement for the Use of Animals in Ophthalmic and Visual Research. They were housed in an indoor environment with a 12 h light/12 h dark cycle, a temperature of 24 ± 2°C, a luminance of approximately 100–200 lux, and free access to food and water. The animal procedures were approved by the Institutional Animal Care and Use Committee of Central South University (Approval No. 2020sydw0077).

### Induction of mouse FDM

Induction of FDM in mice was performed following the procedures described in previous studies (Schaeffel et al., 2004; Wu et al., 2015) with minor modifications. Briefly, on the day of the experiment (Postnatal Day 21–24, weight 10–15 g), male C57BL/6J mice were anesthetized by an intraperitoneal injection of ketamine (90 mg/kg) and xylazine (10 mg/kg), and diffuser eye patches were attached to the skin surrounding the right eye. The diffuser eye patch was made in the laboratory from a plastic tube bottom (diameter: 7.5 mm) mounted on a matching soft latex ring. The eye diffuser was first glued to the periorbital skin around the right eye and then fixed with six to eight stitches (Prolene suture; size 4–0). TobraDex ophthalmic ointment (Alcon, United States) was applied to the eye to protect the cornea from drying. Collars made from plastic foils (outer diameter: 5.5–6.5 cm, inner diameter: 1.0 cm) were fitted around the neck to prevent the mice from removing their diffusers. Food pellets were placed on the floor of the cage to make eating easier. Mice wearing the diffusers were housed in groups of five to six in transparent plastic cages under 12:12 h light-dark conditions (approximately 200 lux illuminance) for 28 days. They were checked every day to ensure the attachment of the diffuser to the eye. A dropped or loose diffuser was reattached. Mice with cataracts or corneal opacity were excluded from the experiments.

### Assessment of refraction and axial length

The diffusers were removed after 28 days of FDM treatment, and both eyes were refracted within the same day. The mice were intraperitoneally anesthetized as previously described. One drop of compound tropicamide solution (Santen Pharmaceutical Co., Ltd., JP) was instilled into each eye to ensure a pupil diameter of 1.5 mm. Full pupil dilatation took several minutes. To avoid cataract formation during anesthetization, the mice were refracted immediately (within minutes). The mice were examined using cycloplegic streak retinoscopy by an experienced optometrist. An interocular refractive difference greater than 5 diopters (D) was considered an indicator of successful induction of FDM, and successful models were used in the subsequent experiments.

**FIGURE 1:**
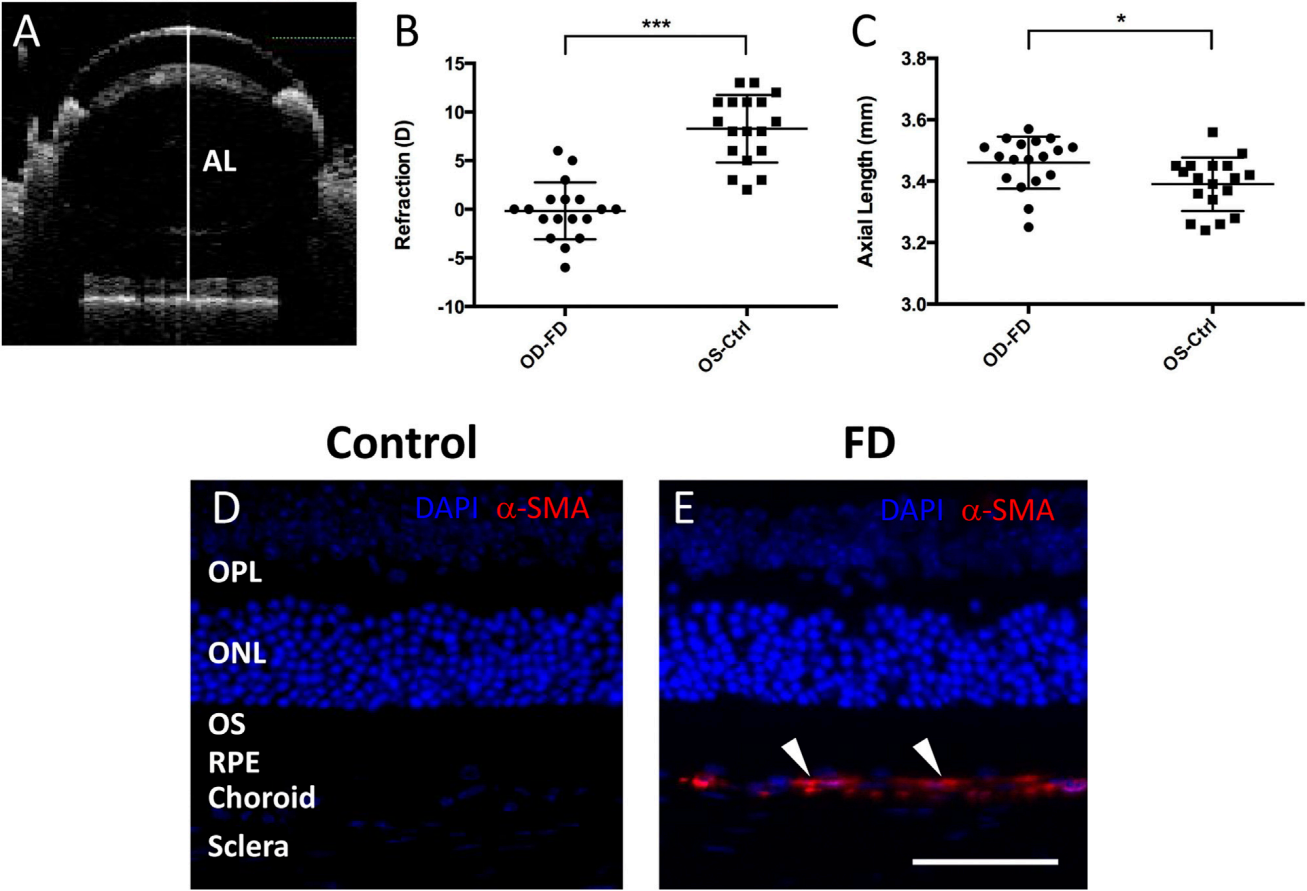
Assessment of FDM model mice. **(A)** Representative SD-OCT images of the axial length (AL) in mice. The white line indicates the ocular AL. After 4 weeks of form deprivation, FDM eyes (OD-FD) display significant myopic refraction **(B)** and significant increases in AL **(C)** compared to fellow controls (OS-Ctrl). Immunostaining for α-SMA (*red, arrowhead*) showed greater expression in the scleral and choroidal layers of FDM mouse eyes **(E)** than in those of control eyes **(D)** on Day 28 of form deprivation induction. DAPI (*blue*).

The axial length of the mice was measured with spectral domain-optical coherence tomography (SD-OCT) under light anesthesia (Banerjee et al., 2020). The anesthetized mouse was placed in front of the light source (Visante OCT 1000, Carl Zeiss Meditec Inc., Dublin, California, United States). The cornea was hydrated with normal saline. The reference arm and focus dial were adjusted simultaneously to a point at which all structures of the eye were in focus. Alignment was confirmed by viewing the radial image of the surface of the eye and adjusting the light source for the central reflection along the horizontal and vertical optical meridians (Figure 1A). Each scan contained an average of 5 images. To measure AL, calipers were placed from the cornea to the retinal pigment epithelium (RPE) border by ImageJ software.

### RNA extraction and sequencing

High-throughput sequencing was performed on the mouse retinas (Majorbio Bio-Pham Technology Co., Shanghai, China). There were 12 samples (6 FDM and 6 fellow eye controls), and each contained 3 retinas (total of 18 mice) to ensure that enough RNA was collected (Supplementary Figure.S1). Retinas were collected after 4 weeks of FDM induction. Total RNA was extracted from the retinas using TRIzol^®^ Reagent (Invitrogen, Carlsbad, CA, United States), and genomic DNA was removed by DNase I RNase-free (Takara). The contamination or degradation of RNA was examined by agarose gel (1%) electrophoresis, and the concentration was measured using a NanoDrop-2000 (Thermo Scientific, Wilmington, DE, United States). RNA integrity was then assessed using a 2100 Bioanalyzer (Agilent Technologies, Santa Clara, CA, United States). Only high-quality RNA samples (OD260/280 = 1.8∼2.2, OD260/230 ≥ 2.0, RIN≥7, 28S:18S ≥ 1.0, >5 μg) were used to construct a sequencing library. Ribosomal RNA depletion was performed using a Ribo-Zero Magnetic Kit (Epicentre Biotechnologies, Madison, WI, United States). A stranded RNA-seq transcriptome library was prepared with a TruSeq™ Stranded Total RNA Kit (Illumina, San Diego, CA, United States). In addition, 3 μg of total RNA was ligated with sequencing adapters with a TruSeq™ Small RNA Sample Prep Kit (Illumina, San Diego, CA, United States). Subsequently, cDNA was synthesized by reverse transcription and amplified with 12 PCR cycles to produce the library. After quantification, the RNA-seq library was sequenced with the HiSeq X Ten (Illumina, San Diego, CA, United States).

### Analysis of sequencing data

The raw paired-end reads were trimmed and quality-controlled with SeqPrep (https://github.com/jstjohn/SeqPrep) and Sickle (https://github.com/najoshi/sickle). The clean reads were aligned to a mouse reference genome (GRCm38.p6) using HISAT2 (V2.1.0) and using bowtie2 (V2.2.9). The mapped reads of each sample were assembled by StringTie (V1.3.3b) in a reference-based approach. Finally, assembled transcripts were annotated by Cuffcompare program from the Cufflinks (V2.2.1).

### Identification of lncRNAs

Known lncRNAs were identified by alignment of the transcripts to the existing reference genome and reported lncRNA sequences in lncRNA-related databases, including NONCODE, Ensembl, NCBI, UCSC, LncRNAdb, GENCODE, GREENC, and LncRNA Disease. Novel lncRNAs were selected step-by-step with criteria. According to the definition and features of lncRNAs, the exclusion criteria for the transcripts were 1) overlapping with known protein-coding genes on the same strand, 2) a fragment count ≤3, 3) a length shorter than 200 nt, 4) an open reading frame (ORF) longer than 300 nt, and 5) an exon number less than 2. Next, the Coding Potential Calculator (CPC), Coding-Non-Coding index (CNCI), Coding Potential Assessment Tool (CPAT), and Pfam Scan were used to filter transcripts with coding potential. The remaining transcripts were considered reliably expressed lncRNAs. Using Cuffcompare in Cufflinks, lncRNAs were classified into intergenic, intronic, and antisense lncRNAs.

### Expression analysis of lncRNAs and mRNAs

The quantitative expression of both lncRNAs and mRNAs in each sample was calculated in transcripts per kilobase of exon model per million mapped reads (TPM). lncRNAs with |log2(FDM/ctrl)| >1 and FDR (Q value) < 0.05 as determined by EdgeR were considered significantly differentially expressed (DE) transcripts. Volcano plots and hierarchical clustering were used to analyze the DE lncRNAs and mRNAs identified between FDM and fellow control retinas. The predicted potential target genes whose loci were within a 10-kb window upstream or downstream of the given aberrantly expressed lncRNA were considered cis-regulated genes. Other genes in the co-expression network were identified as trans-regulated according to complementary base pairing by LncTar. Also the intaRNA (V2.3.1), RNAplex, RIblast (V1.1.3) were used to predict the target genes. The expressed correlation was calculated between lncRNAs and target genes, and a Pearson correlation coefficient >0.9 identified the target genes.

### Quantitative real-time polymerase chain reaction (qRT–PCR)

The expression of ten lncRNAs in the retinas of four mice (control fellow retinas, *n* = 4; FDM retinas, *n* = 4) was assessed using qRT–PCR to verify the accuracy of the high-throughput sequencing results. Eyes were enucleated, and the retinas were immediately dissected. Total RNA was extracted from samples by using TRIzol^®^ Reagent (Invitrogen, Carlsbad, CA, United States), and cDNA was synthesized by using a miScript II RT Kit (Qiagen, Hilden, Germany). Real-time PCR was performed with a miScript SYBR^®^ Green PCR Kit (Qiagen, Hilden, Germany) using a 7500 FAST real-time PCR system (Applied Biosystems, Foster City, CA, United States). The expression of lncRNAs was calculated by the 2^−ΔΔCt^ method. A two-tailed Student**’**s t test was used to compare lncRNA expression between samples from the fellow eyes and those from the FDM eyes in 3 experimental replicates. The forward and reverse primers for lncRNAs are shown in Supplementary Table S1.

### Immunofluorescence

Eyes were enucleated and fixed in FAS eyeball fixative solution (G1109-100ML, Servicebio, Wuhan, China) at 4°C for 24 h. The tissues were cryoprotected in 20% sucrose in PBS and embedded in optimal cutting temperature (O.C.T.) compound (Tissue-Tek; Sakura Finetek, Torrance, CA, United States). Twenty-micrometer cryosections were first blocked with serum and immunolabeled with a primary rabbit IgG anti-SMA mAb (1:200; Abcam, Temecula, CA, United States), a primary rabbit IgG anti-RBMPS mAb (1:100; GTX118619 GeneTex, CA, United States), or a primary rabbit IgG anti-calbindin mAb (1:100; Bioworld, Nanjing, China) at 4°C overnight. Then, the sections were reacted with the corresponding fluorescein isothiocyanate-conjugated secondary antibody and finally evaluated by fluorescence microscopy. The slides were stained with DAPI (G1012, Servicebio) for mounting.

### RNA fluorescence *in situ* hybridization (FISH)

After 4 weeks of FDM induction, mice were killed, and the eyes were enucleated. The eyes were fixed in FAS eyeball fixative solution (G1109-100ML, Servicebio, Wuhan, China) for more than 24 h. The eyes were dehydrated and embedded in paraffin. The paraffin-embedded eyes were sectioned at 5-μm thickness and baked on microscope slides in a hybridization oven at 62°C for 2 h before *in situ* hybridization. The probes labeled with DIG for lncRNA XR_384718.4 (Gm35369) are shown in Supplementary Table S1. The sections were washed with PBS and blocked with rabbit serum blocking buffer after prehybridization and hybridization. Next, the sections were incubated with mouse anti-DIG-HRP (Jackson ImmunoResearch Labs Inc., United States) for 40 min. After two washes with PBS for 5 min, fresh tyramide signal amplification chromogenic reagent (G3025, Servicebio) was used for the chromogenic reaction for 5 min. The sections were stained with DAPI (G1012, Servicebio) for 8 min and mounted. Photographs were obtained with a fluorescence microscope (Nikon Eclipse CI, Japan).

### Statistical analysis

The data are reported as the mean ± standard error of the mean (SEM). Graphs were constructed using GraphPad Prism 9.0 software. Statistical Program for the Social Sciences 20.0 software (IBM SPSS Inc., New York, NY) was used for statistical analysis of the biometric parameters and qRT–PCR results. The ocular biometric parameters in the myopia-induced right eyes (OD) and the untreated contralateral left fellows (OS) were compared using the paired t test. The sample sizes are reported in the results. Overall comparisons of these indices in the right eyes among the experimental groups were performed with two-way analysis of variance (ANOVA) or one-way ANOVA, and pairwise comparisons were performed with Tukey’s post hoc test. *p* values <0.05 were considered to indicate significance.

## Results

### Establishment and analysis of FDM mice 

Visual form deprivation in C57BL/6J mice was started at approximately 3 weeks (P21, weight 12.82 ± 2.22 g, *n* = 18). After 28 days of form deprivation in photopic conditions, a myopic shift in ocular measurements was observed compared to that in the left control eyes (Figure 1B). The refraction (in diopters, D) in deprived eyes (−0.17 ± 2.94 D) was shifted toward myopia compared to that in the controls (+8.28 ± 3.48 D); the interocular differences in refraction between the right and left eyes (OD-OS, −8.44 ± 4.93 D, *p* < 0.001) were statistically significant. The axial length (mm) was also elongated in the deprived eyes (3.46 ± 0.09 mm) relative to that in the controls (3.39 ± 0.09 mm), with a significant interocular difference (OD-OS, 0.07 ± 0.09 mm, *p* < 0.05) (Figure 1C). To further confirm the characteristics of FDM eyes, the expression of alpha-smooth muscle actin (α-SMA), a myofibroblast marker, was examined with immunostaining in the retina and sclera. In accordance with previous studies (Wu et al., 2015; Yuan et al., 2018), α-SMA was more highly expressed in the FDM sclera and choroid areas than in these areas in the control fellows after 4 weeks of induction (Figures 1D,E). These results suggested the development of significant FDM in the goggled mouse eyes.

### Sequencing data summary

Libraries were constructed from retinal tissue samples from FDM eyes (*n* = 6; each sample consisted of three retinas from three FDM eyes to ensure that the RNA amount was sufficient) and control fellows (*n* = 6; each sample consisted of three retinas from the control fellow eyes of the FDM eyes) and subjected to sequencing analysis. RNA-seq yielded 501,329,752 and 547,167,536 raw reads from the FDM and control groups, respectively. Low-quality reads were filtered from the raw reads, and high-quality clean reads and clean bases were obtained. In total, 498,008,508 and 543,470,042 clean reads were retained for the FDM and control groups, respectively. The Q20 and Q30 quality scores of the clean data were higher than 90%, indicating the reliability of the RNA sequencing results. The clean reads were mapped to a mouse reference genome (GRCm38. p6, Ensembl) with a total mapping percentage ranging from 96.29 to 97.15%. Detailed data on the quality results are shown in Supplementary Table S2.

### Identification and classification of lncRNAs in the retinas of mice

**FIGURE 2:**
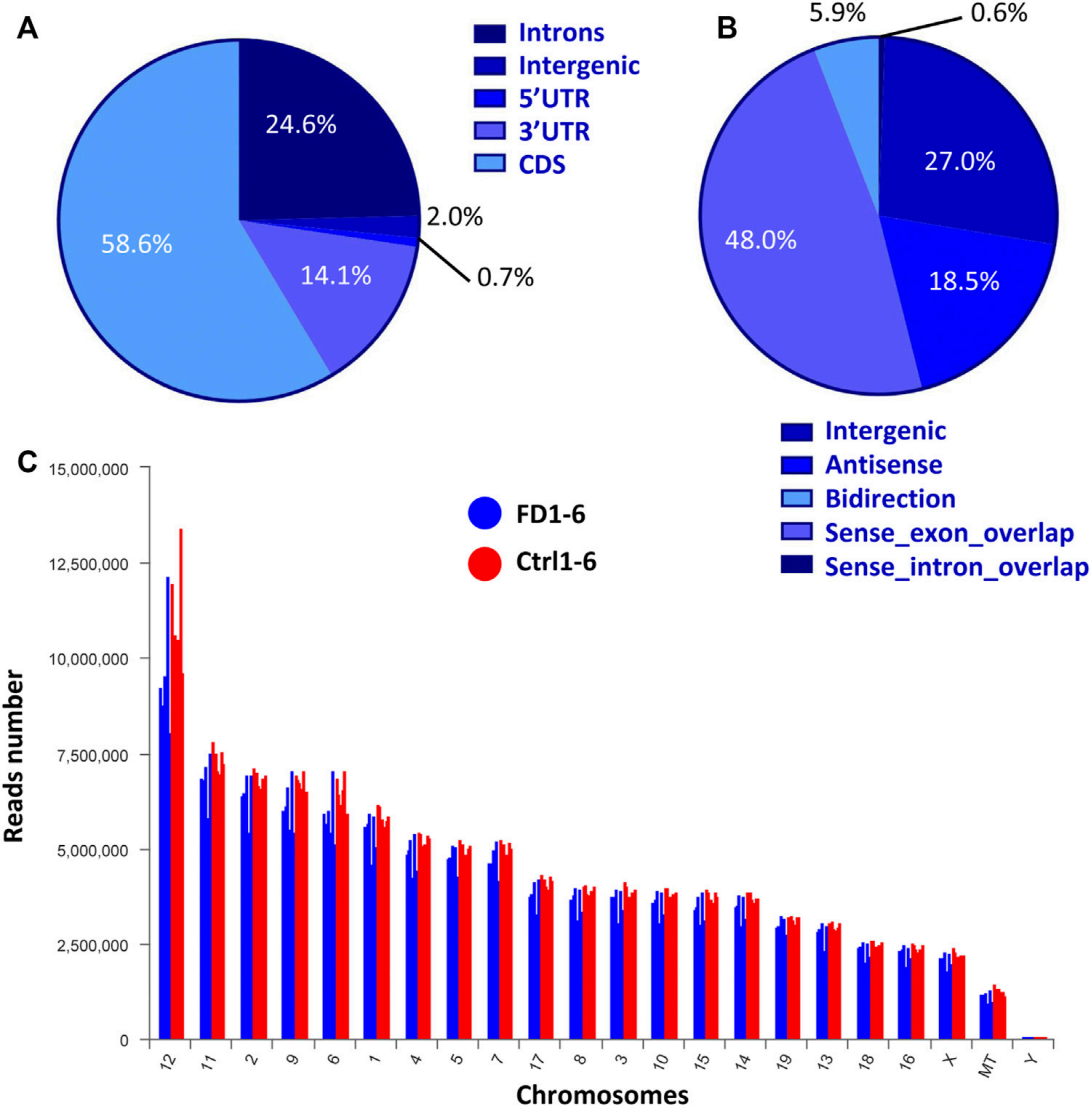
Expression characteristics of lncRNAs from both FDM and control eyes. **(A)** Percentages of reads mapped to genomic regions. **(B)** Percentages of lncRNAs classified into different groups. **(C)** Chromosomal distribution of lncRNA numbers.

According to the mouse reference genome and related databases (NONCODE, Ensembl, and NCBI), 19,443 known lncRNAs were identified. Filtering and overlapping analyses in four programs (PfamScan, CPC, CPAT, and CNCI) identified a total of 561 novel lncRNAs (Supplementary Figure S2). Mapping of the reads to genomic regions with RSeQC-2.3.6 revealed the distributions of the lncRNAs from both FDM and control eyes in five areas: the 5′UTR (0.81%), intergenic regions (1.97%), the 3′UTR (14.06%), introns (24.57%), and the coding sequence (CDS, 58.58%) (Figure 2A). Based on the relative chromosomal position of the coding gene, the novel lncRNAs were classified into five categories: 5 were sense intronic overlapping lncRNAs (0.6%), 234 were intergenic lncRNAs (27.0%), 160 were antisense lncRNAs (18.5%), 416 were sense exonic overlapping lncRNAs (48.0%), and 51 were bidirectional lncRNAs (5.9%) (Figure 2B). Chromosomal distribution analysis of the lncRNAs showed that chromosomes 12, 11, 2 and 9 contained relatively higher amounts of lncRNAs than the other chromosomes (Figure 2C).

### Differential expression patterns of lncRNAs and mRNAs in FDM

**FIGURE 3:**
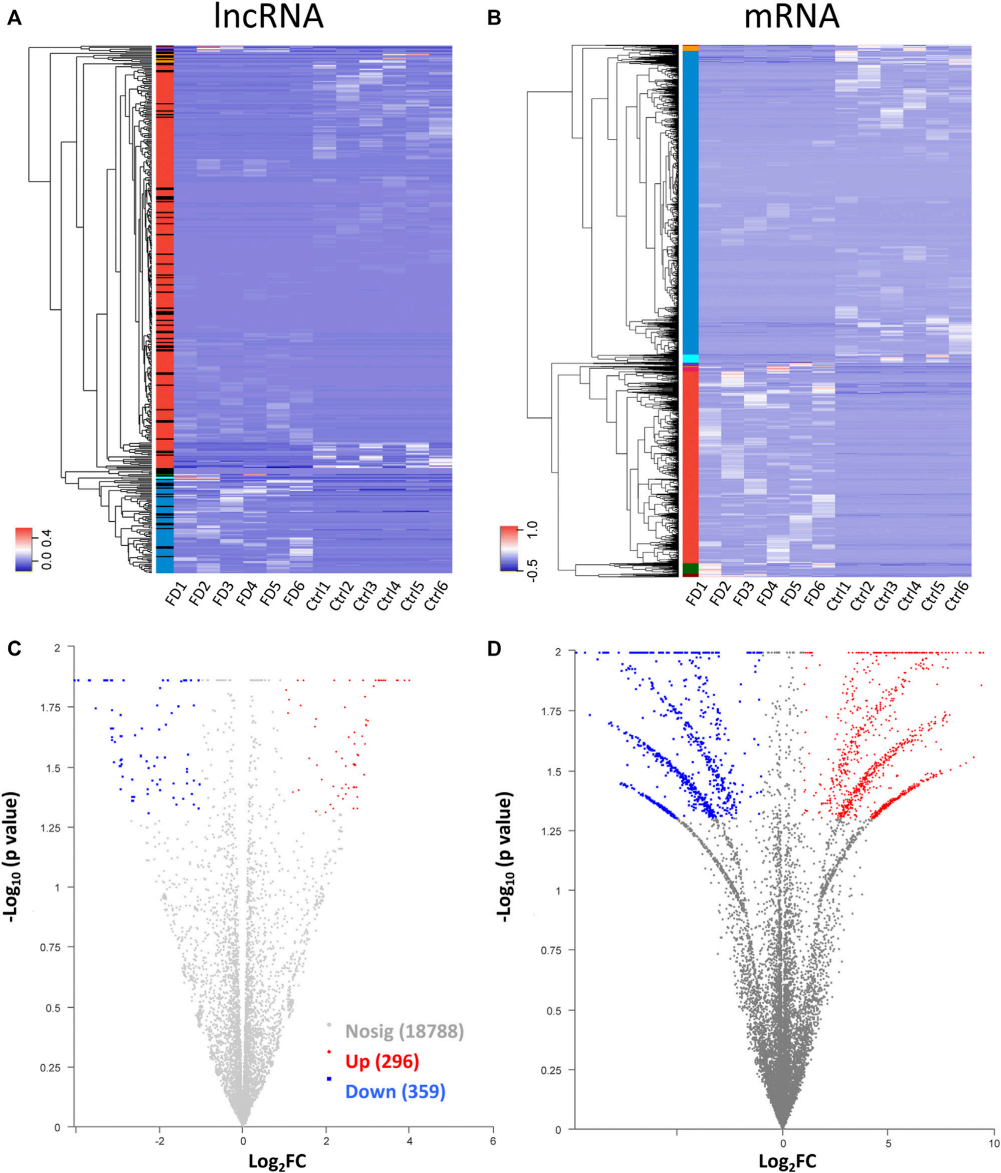
Heatmap for hierarchical clustering of lncRNAs **(A)** and mRNAs **(B)** from 12 samples (six for FDM and 6 for control). The colors in the panel represent the relative expression levels: blue and red represent low and high expression levels, respectively. Volcano plot of lncRNAs **(C)** and mRNAs **(D)**. Red/blue dots represent significantly up//downregulated RNAs (FC ≥ 2.0, *p* < 0.05). Gray indicates no differential expression.

Among the 20,309 lncRNAs (19,443 from reference databases, 866 of novel) obtained from high-throughput sequencing, hierarchical clustering analysis showed that there were 655 differentially expressed lncRNAs between the FDM and control retinas, of which 296 were upregulated and 359 were downregulated. The top 20 differentially upregulated (Table 1) and downregulated lncRNAs (Table 2) between the FDM and control retinas, such as XR_003956022.1, NR_045075.1, and Oip5os1, are listed according to the statistical significance (*p* value) and log2FC. Among the 19,137 mRNAs (18,580 from reference databases, 557 of novel) obtained from RNA-seq, there were 478 differentially expressed mRNAs (206 upregulated and 272 downregulated) between the FDM and control. The top 20 differentially up- and downregulated mRNAs with the largest fold changes are also displayed (Tables 3, 4). Heatmaps and volcano plots were used to visualize the differentially expressed lncRNAs and mRNAs between the two groups (Figure 3).

**TABLE 1:** Top 20 Up-regulated LncRNAs (*p* < 0.05) Between FDM and Fellow Groups. Log2FC, Log2FC of (FDM retina/fellow control retina).

Transcript ID	Gene ID	Gene Name	Gene description	Log2FC	*p* value
XR_003956022.1	ENSMUSG00000100783	2310047D07Rik	RIKEN cDNA 2310047D07 gene	7.793	9.66E-03
NR_045075.1	ENSMUSG00000086363	A330102I10Rik	RIKEN cDNA A330102I10 gene	7.379	1.76E-03
chr4:21834991-21837868	ENSMUSG00000040455	Usp45	ubiquitin specific petidase 45	7.182	2.96E-03
ENSMUST00000147425	ENSMUSG00000085438	Oip5os1	Opa interacting protein 5, opposite strand 1	6.852	6.60E-03
chr16:32388756-32392334	ENSMUSG00000053774	Ubxn7	UBX domain protein 7	6.666	1.29E-02
chr6:92167828-92169115	ENSMUSG00000005893	Nr2c2	nuclear receptor subfamily 2, group C, member 2	6.249	6.69E-03
XR_871884.3	ENSMUSG00000112110	Gm15608	predicted gene 15608	6.235	1.26E-03
ENSMUST00000181960	ENSMUSG00000097290	1300002E11Rik	RIKEN cDNA 1300002E11 gene	6.172	4.21E-03
XR_004934313.1	ENSMUSG00000109233	Gm44866	predicted gene 44866	6.145	1.48E-02
chr7:101793411-101795506	ENSMUSG00000001829	Clpb	ClpB caseinolytic peptidase B	6.026	1.58E-02
chr13:114155322-114157022	ENSMUSG00000042348	Arl15	ADP-ribosylation factor-like 15	5.933	3.65E-03
chr17:88487362-88490321	ENSMUSG00000034998	Foxn2	forkhead box N2	5.837	1.58E-02
XR_004941494.1	ENSMUSG00000104178	Gm9916	predicted gene 9916	5.645	4.91E-03
XR_004940873.1	ENSMUSG00000086405	9330198N18Rik	RIKEN cDNA 9330198N18 gene	5.637	4.93E-03
chr1:34446795-34449308	ENSMUSG00000026127	Imp4	IMP4, U3 small nucleolar ribonucleoprotein	5.481	1.82E-02
ENSMUST00000128131	ENSMUSG00000086290	Snhg12	small nucleolar RNA host gene 12	5.464	9.49E-03
XR_381591.4	ENSMUSG00000112412	Gm35239	predicted gene, 35239	5.380	1.93E-02
ENSMUST00000152024	ENSMUSG00000086587	Gm11837	predicted gene 11837	5.352	2.69E-03
XR_001783522.3	ENSMUSG00000085317	Gssos2	glutathione synthase, opposite strand 2	5.169	5.48E-03
XR_880469.2	ENSMUSG00000090006	Gm16227	predicted gene 16227	5.166	2.14E-02

**TABLE 2:** Top 20 down-regulated LncRNAs (*p* < 0.05) between FDM and fellow groups.

Transcript ID	Gene ID	Gene Name	Gene description	Log2FC	*p* value
chr6:13086757-13089260	ENSMUSG00000029571	Tmem106b	transmembrane protein 106B	−6.240	1.57E-02
XR_004935657.1	ENSMUSG00000103640	Gm31406	predicted gene, 31406	−6.161	7.98E-03
chr9:110981867-110984062	ENSMUSG00000032495	Lrrc2	leucine rich repeat containing 2	−6.031	4.86E-03
XR_003955073.1	ENSMUSG00000087366	Junos	jun proto-oncogene, opposite strand	−5.990	2.54E-04
XR_386631.3	ENSMUSG00000117692	Gm50114	predicted gene, 50114	−5.928	1.73E-02
ENSMUST00000126380	ENSMUSG00000086290	Snhg12	small nucleolar RNA host gene 12	−5.873	2.33E-03
NR_040262.1	ENSMUSG00000044471	Lncpint	Trp53 induced transcript	−5.825	8.89E-03
XR_881968.1	ENSMUSG00000108711	Gm38991	predicted gene, 38991	−5.806	1.40E-02
XR_871885.3	ENSMUSG00000112110	Gm15608	predicted gene 15608	−5.731	4.93E-03
XR_003948926.1	ENSMUSG00000112110	Gm15608	predicted gene 15608	−5.723	1.15E-03
ENSMUST00000179924	ENSMUSG00000079179	Rab10os	RAB10, RAS oncogene family, opposite strand	−5.656	4.90E-03
XR_004934966.1	ENSMUSG00000110559	Gm26843	predicted gene, 26843	−5.612	1.04E-02
chr8:61504964-61506681	ENSMUSG00000031641	Cbr4	carbonyl reductase 4	−5.567	4.08E-03
XR_004942277.1	ENSMUSG00000086953	Aknaos	AT-hook transcription factor, opposite strand	−5.489	1.98E-02
chr7:113928073-113932081	ENSMUSG00000038156	Spon1	spondin 1, (f-spondin) extracellular matrix protein	−5.482	2.03E-02
ENSMUST00000238598	ENSMUSG00000097129	4930507D05Rik	RIKEN cDNA 4930507D05 gene	−5.468	6.20E-03
ENSMUST00000238778	ENSMUSG00000026736	4930426L09Rik	RIKEN cDNA 4930426L09 gene	−5.462	1.07E-02
chr1:15716879-15719068	ENSMUSG00000092083	Kcnb2	potassium voltage gated channel, Shab member 2	−5.370	1.19E-02
chr18:36299103-36299691	ENSMUSG00000110185	Igip	IgA inducing protein	−5.364	1.44E-02
XR_004941495.1	ENSMUSG00000104178	Gm9916	predicted gene 9916	−5.295	6.54E-03

**TABLE 3:** Top 20 upregulated mRNA (*p* < 0.05) between FDM and fellow groups.

Transcript ID	Gene ID	Gene Name	Gene description	Log2FC	*p* value
ENSMUST00000021662	ENSMUSG00000021236	Entpd5	ectonucleoside triphosphate diphosphohydrolase 5	9.540	4.56E-03
ENSMUST00000211820	ENSMUSG00000037270	4932438A13Rik	RIKEN cDNA 4932438A13 gene	9.436	1.39E-02
ENSMUST00000101375	ENSMUSG00000057113	Npm1	nucleophosmin 1	9.386	9.60E-04
ENSMUST00000095172	ENSMUSG00000034390	Cmip	c-Maf inducing protein	9.337	3.42E-03
ENSMUST00000097785	ENSMUSG00000026131	Dst	dystonin	9.328	2.92E-03
ENSMUST00000228412	ENSMUSG00000002496	Tsc2	TSC complex subunit 2	9.061	2.79E-02
ENSMUST00000132158	ENSMUSG00000026696	Vamp4	vesicle-associated membrane protein 4	8.938	8.63E-03
ENSMUST00000082170	ENSMUSG00000074505	Fat3	FAT atypical cadherin 3	8.701	1.89E-03
ENSMUST00000117805	ENSMUSG00000048240	Gng7	guanine nucleotide binding protein, gamma 7	8.681	2.95E-02
ENSMUST00000068367	ENSMUSG00000032396	Dis3l	DIS3 like exosome 3′–5′ exoribonuclease	8.422	3.03E-02
ENSMUST00000227200	ENSMUSG00000048038	Ccdc187	coiled-coil domain containing 187	8.308	3.35E-03
ENSMUST00000204198	ENSMUSG00000001632	Brpf1	bromodomain and PHD finger containing, 1	8.063	9.12E-03
ENSMUST00000238849	ENSMUSG00000068876	Cgn	cingulin	7.970	3.14E-02
ENSMUST00000061970	ENSMUSG00000031337	Mtm1	X-linked myotubular myopathy gene 1	7.904	1.85E-02
ENSMUST00000125774	ENSMUSG00000026426	Arl8a	ADP-ribosylation factor-like 8A	7.899	4.95E-03
ENSMUST00000233357	ENSMUSG00000117098	Gm49909	predicted gene, 49909	7.841	1.19E-02
ENSMUST00000111372	ENSMUSG00000040687	Madd	MAP-kinase activating death domain	7.786	1.98E-02
ENSMUST00000163854	ENSMUSG00000026074	Map4k4	mitogen-activated protein 4 kinase 4	7.753	1.08E-02
ENSMUST00000144936	ENSMUSG00000079020	Slc45a4	solute carrier family 45, member 4	7.732	1.86E-02
ENSMUST00000166592	ENSMUSG00000031691	Tnpo2	transportin 2 (importin 3, karyopherin beta 2b)	7.729	1.01E-02

**TABLE 4:** Top 20 downregulated mRNA (*p* < 0.05) between FDM and fellow groups.

Transcript ID	Gene ID	Gene Name	Gene description	Log2FC	*p* value
ENSMUST00000137823	ENSMUSG00000056342	Usp34	ubiquitin specific peptidase 34	−9.792	3.21E-03
ENSMUST00000085044	ENSMUSG00000006676	Usp19	ubiquitin specific peptidase 19	−9.352	5.12E-03
ENSMUST00000003191	ENSMUSG00000024070	Prkd3	protein kinase D3	−9.124	1.86E-02
ENSMUST00000234851	ENSMUSG00000061130	Ppm1b	protein phosphatase 1B, beta isoform	−8.760	1.08E-02
ENSMUST00000107417	ENSMUSG00000042626	Shc1	src homology 2 transforming protein C1	−8.704	3.18E-03
ENSMUST00000019246	ENSMUSG00000019102	Aldh3a1	aldehyde dehydrogenase family 3, A1	−8.484	9.23E-03
ENSMUST00000181981	ENSMUSG00000045659	Plekha7	pleckstrin homology family A member 7	−8.459	8.64E-04
ENSMUST00000084301	ENSMUSG00000028649	Macf1	microtubule-actin crosslinking factor 1	−8.457	3.38E-03
ENSMUST00000238066	ENSMUSG00000052387	Trpm3	transient receptor potential channel M3	−8.380	9.90E-03
ENSMUST00000212478	ENSMUSG00000036180	Gatad2a	GATA zinc finger domain containing 2A	−8.264	5.04E-03
ENSMUST00000099149	ENSMUSG00000025453	Nnt	nicotinamide nucleotide transhydrogenase	−8.218	2.02E-02
ENSMUST00000112990	ENSMUSG00000000266	Mid2	midline 2	−8.145	2.00E-02
ENSMUST00000076140	ENSMUSG00000033577	Myo6	myosin VI	−8.088	2.07E-02
ENSMUST00000095012	ENSMUSG00000028883	Sema3a	semaphorin 3A	−7.839	2.08E-02
ENSMUST00000169854	ENSMUSG00000056917	Sipa1	signal-induced proliferation associated 1 Symbol; Acc:MGI:107576	−7.783	2.13E-02
ENSMUST00000224209	ENSMUSG00000037824	Tspan14	tetraspanin 14	−7.778	1.71E-03
ENSMUST00000113530	ENSMUSG00000030087	Klf15	Kruppel-like factor 15	−7.753	1.14E-03
ENSMUST00000001043	ENSMUSG00000001017	Chtop	chromatin target of PRMT1	−7.696	3.60E-02
ENSMUST00000114617	ENSMUSG00000031337	Mtm1	X-linked myotubular myopathy gene 1	−7.648	1.21E-02
ENSMUST00000164039	ENSMUSG00000043531	Sorcs1	sortilin-related VPS10 domain containing receptor 1	−7.645	3.60E-02

### Gene ontology and kyoto encyclopedia of genes and genomes analysis

**FIGURE 4:**
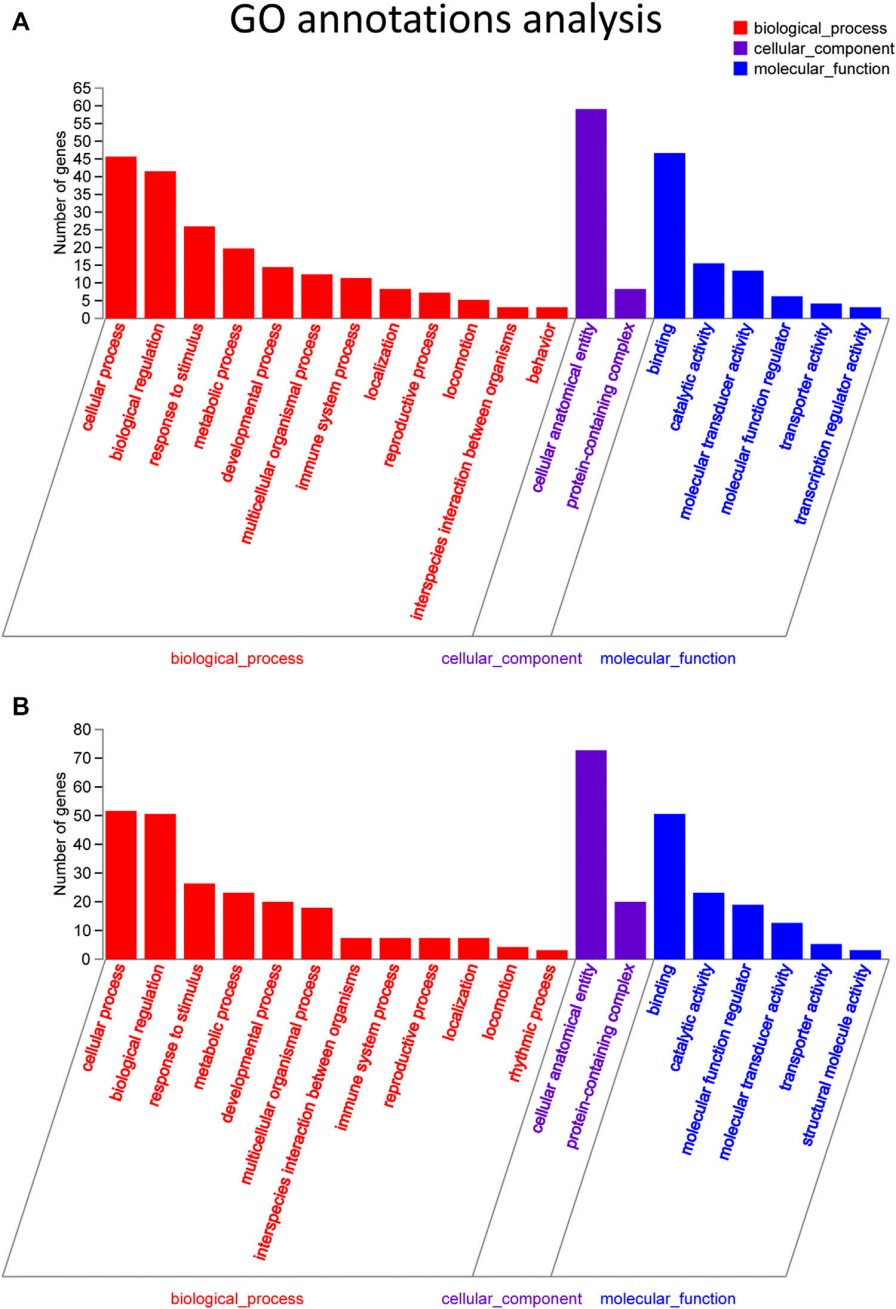
GO pathway analysis in FDM retinas. **(A)** GO analysis of significantly upregulated mRNAs. The top 20 GO terms in the biological process (BP), cellular component (CC) and molecular function (MF) categories are shown for the upregulated mRNAs. **(B)** GO analysis of significantly downregulated mRNAs. Y-axis, number of genes included in a single annotation; X-axis, GO pathway terms. The circle size represents the gene number. The FDR value is indicated by the color gradient. FDR <0.05 indicates significant enrichment of the functional pathway.

Target genes of the differential lncRNAs were predicted by bioinformatics approaches, and the prediction results are illustrated in Supplementary Table S3. The differentially expressed mRNAs underwent GO and KEGG enrichment analyses. GO enrichment analysis examined the gene functions in three categories: the cellular component (CC), biological process (BP), and molecular function (MF) categories. The top 20 enriched GO terms of the significantly upregulated mRNAs are presented and included the cellular process (ontology: BP, GO: 0009987), cellular anatomical entity (ontology: CC, GO: 0110165), and binding (ontology: MF, GO: 0005488) terms (Figure 4A). The downregulated mRNAs were related to some different terms, such as rhythmic process (ontology: BP, GO: 0048511) and structural molecule activity (ontology: MF, GO: 0005198) (Figure 4B).

**FIGURE 5:**
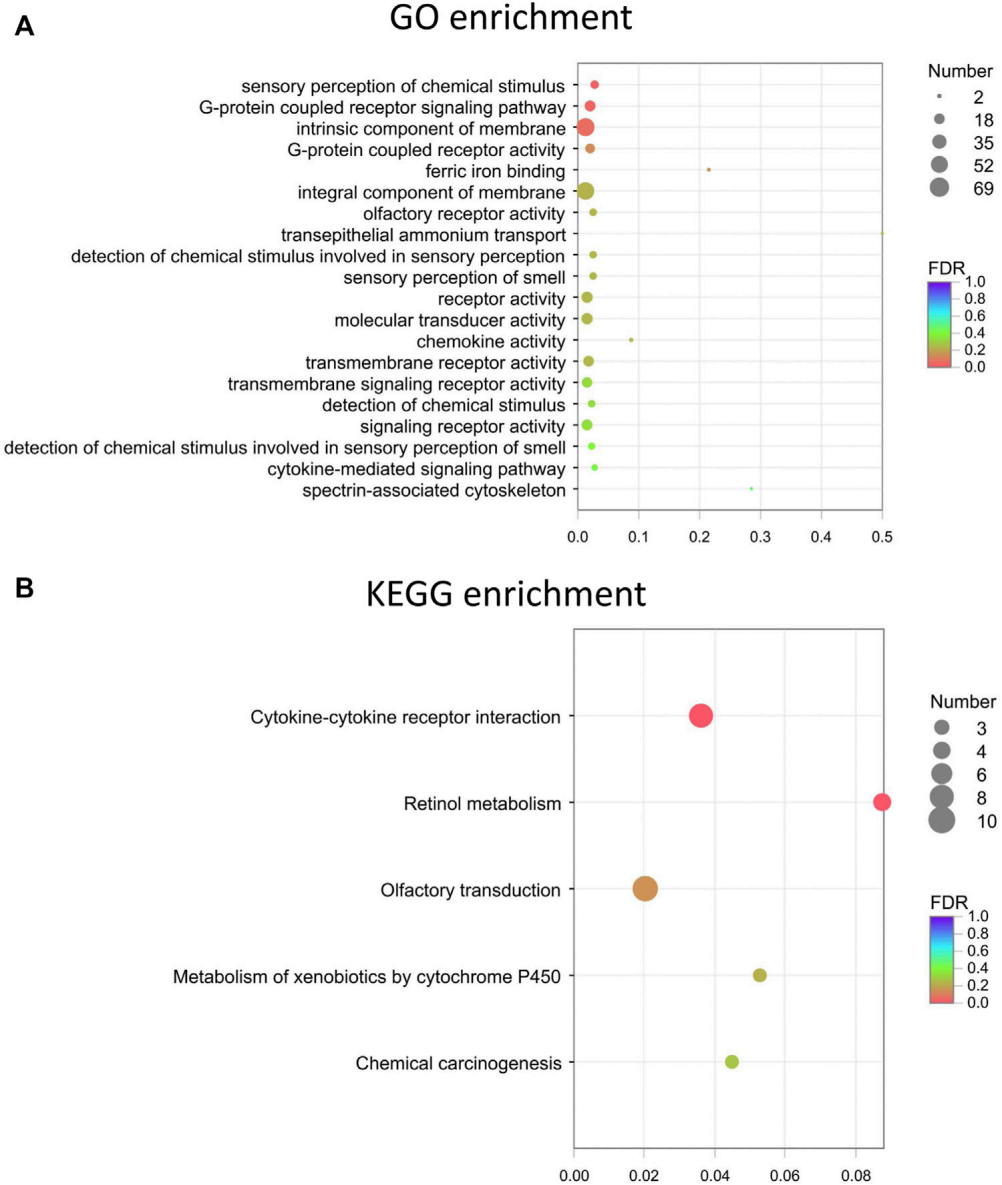
The top 20 GO and KEGG enrichment of the mRNAs. **(A)** Top 20 GO terms for the differentially expressed mRNAs. **(B)** Top KEGG enrichment for the differentially expressed mRNAs. Dot color: towards blue, FDR towards 1.0; towards red, FDR toward 0.0. Dot size: the number of core genes within the pathway.

GO enrichment analyses were performed on the differentially expressed mRNAs. The top 20 GO and KEGG enrichment of the mRNAs are shown in Figures 5A,B, respectively. The terms “sensory perception of chemical stimulus” and “G-protein coupled receptor signaling pathway” were among the top enriched in the GO enrichment analysis (Figure 5A). Multiple pathways, such as cytokine-cytokine receptor interactions, retinol metabolism, olfactory transduction, metabolism of xenobiotics by cytochrome P450, chemical carcinogenesis, tyrosine metabolism, and proteasome, are likely involved in FDM (Figure 5B). The expression levels of altered genes and their related enriched KEGG pathways are illustrated in the KEGG chord plot (Supplementary Figure S3). For example, Ccl21d, Epo, Ccl22, Tnfrsf17, Il22ra1, Pf4, Ccl27a, Cd70, and Il21r were associated with cytokine-cytokine receptor interaction (pathway ID: map04060), while Bco1, Cyp3a13, Rdh9, Cyp2a5, and Adh7 were associated with retinol metabolism (pathway ID: map00860). These results indicate that the differentially expressed retinal lncRNAs participate in a variety of biological mechanisms and are intrinsically associated with form-deprived myopia.

### LncRNA-mRNA coexpression network

**FIGURE 6:**
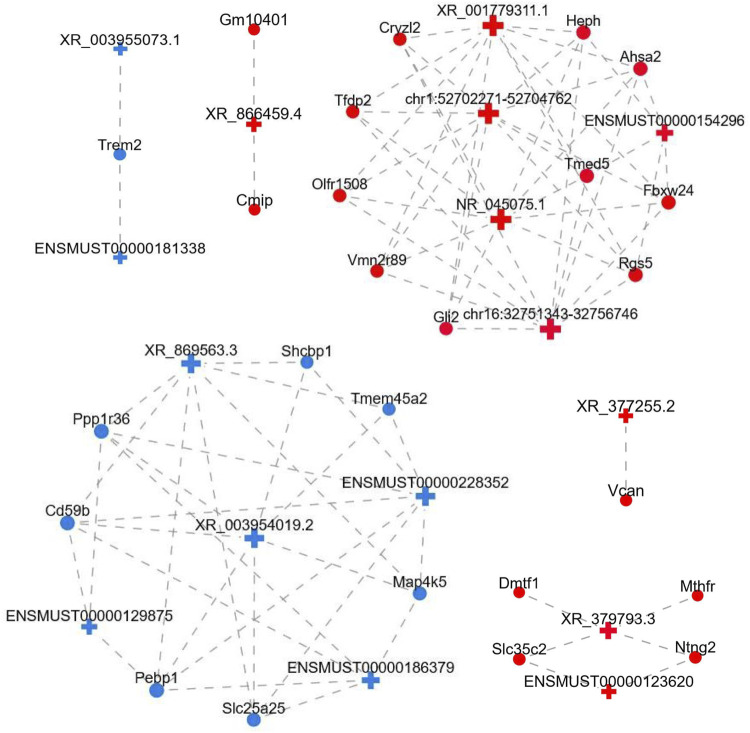
LncRNA-mRNA coexpression network. LncRNAs and mRNAs with Corr >0.95 and *p* value <0.05 were selected to construct the network. The network shows the interactions among the lncRNAs and their potential regulated coding genes. Red, upregulated; blue, downregulated; cross, lncRNA; circle, mRNA; gray dotted line, correlation relationship.

To uncover the possible interactions between lncRNAs and mRNAs in the FDM retina, the lncRNA-mRNA coexpression relationship was identified based on the top differential lncRNAs and mRNAs. After screening (correlation coefficient, Corr >0.95, *p* value <0.05), a lncRNA-mRNA coexpression network, which consisted of 117 nodes (lncRNAs and mRNAs) and 755 edges connecting the nodes, was constructed (Figure 6). The interactive mRNAs included Vcan, Cmip, Trem2, Dmtf1, Cd59b, Shcpb1, Vmn2r89, and others, which might play regulatory roles in myopic biological processes. In particular, the downregulated lncRNA XR_869563.3 and the upregulated lncRNA NR_045075.1 were connected by a large number of mRNAs, which suggests that these dysregulated lncRNAs might be involved in additional functional pathways and mechanisms in the myopic retina.

### Validation of the expression levels of lncRNAs by qRT–PCR

**FIGURE 7:**
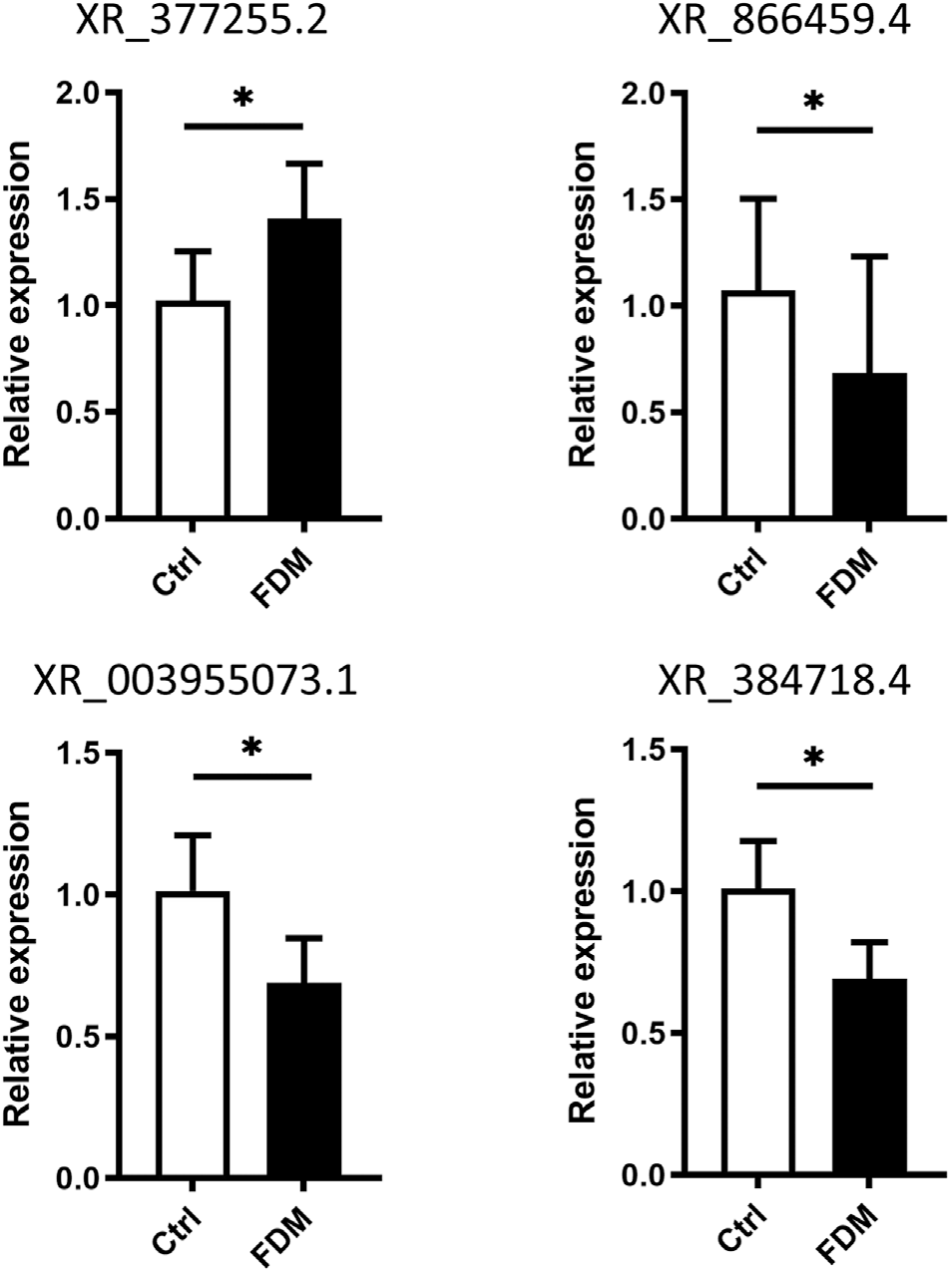
Validation of differential lncRNA expression by qRT–PCR. The relative expression of the lncRNAs XR_377255.2, XR_866459.4, XR_003955073.1, and XR_384718.4 in the retinas from FDM eyes and fellow control eyes is shown. For each group, *n* = 6. *, *p* < 0.05, Student t test.

To verify the expression levels of lncRNAs in myopic retinas, we selected four lncRNAs for qRT–PCR based on the following criteria: A *p* value <0.05 with top-ranked fold change (FC). The LncRNAs were chose for qRT-PCR validation not only based on their expression fold changes, but also according to possible specific interesting correlations, and the lncRNAs records in NCBI databases. For instance, coexpression analysis of XR_377255.2 (log2FC = 5.028) showed correlation with Vcan (Versican), a critical extracellular matrix regulator of immunity and inflammation (Figure 6); while CMIP, C-Maf-inducing protein, was correlated with downregulation of the lncRNA XR_866459.4 (log2FC = 3.430). The XR_003955073.1 (log2FC = −5.990) was in the top 20 down-regulated list. The XR_384718.4 was a *Mus* *musculus* predicted gene (ncRNA), 35369 (Gm35369), transcript variant X2 by NCBI database. It is not in the top down-regulated list but still shows a log2FC of −2.316 (downregulated in myopic retinas as compared to the control). We also hope to test the DE lncRNA with moderate dysregulated expression level. Among the four lncRNAs, XR_377255.2, XR_866459.4 were upregulated, while XR_003955073.1, and XR_384718.4 were downregulated in FDM retinas according to the RNA sequencing data analysis (Supplementary Figure S4; Supplementary Table S4). Increased expression levels of XR_377255.2 (gene name: Gm15411) (*p* = 0.0149) and reduced expression of XR_003955073.1 (gene name: Junos), XR_384718.4 (gene name: Gm35369) and XR_866459.4 (gene name: Gm39857) were observed in FDM mouse retinas (*p* = 0.0451, *p* = 0.0217, and *p* = 0.0423, respectively; Figure 7). Among them, the changes in three (except for XR_866459.4) were consistent with the sequencing results. Thus, we validated the expression changes of XR_377255.2, XR_003955073.1, and XR_384718.4 by qRT-PCR.

### Localization of differentially expressed lncRNAs in the retina by RNA FISH

**FIGURE 8:**
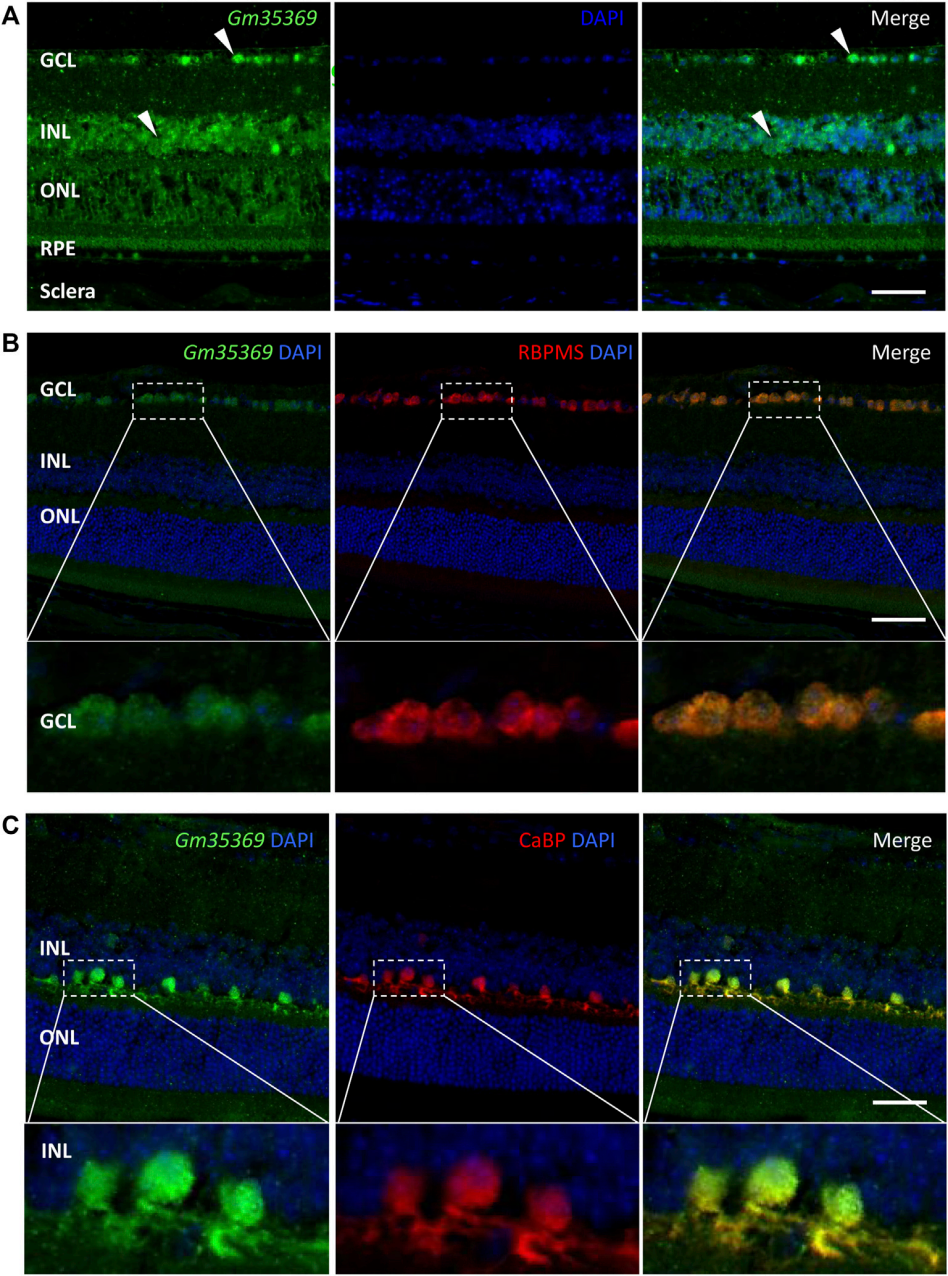
RNA FISH showing localization of the lncRNA Gm35369 in the retina. **(A)** RNA fluorescence *in situ* hybridization showing the localization of Gm35369 in the retina, mostly in the GCL and INL. **(B)** Colocalization of the retinal ganglion cell markers RBPMS (*red*) and Gm35369 (*green*) in the GCL. **(C)** Colocalization of the horizontal cell markers CaBP (*red*) and Gm35369 (*green*). Signals were apparent in both the nucleus and cytoplasm. DAPI (*blue*).

LncRNAs act in different ways to interfere with cellular physiology, depending on their subcellular locations. For the retina, it is also important to identify the layers and cell types in which the targets are located. To further investigate the newly discovered targets in the retina, we conducted a preliminary localization experiment to identify the retinal layers of lncRNAs and their subcellular expression. FISH assay of the lncRNA XR_384718.4 (gene name: Gm35369) in myopic mouse eyecups showed that Gm35369 preferentially localized mostly in the GCL and INL (Figure 8A). Although qRT–PCR showed that it was downregulated in the myopic retina, Gm35369 localized in similar patterns in the retinas of both groups, as the difference between the myopia and control groups was too subtle to be observed by *in situ* hybridization (data not shown). Further colocalization study revealed the overlap between the signal of Gm35369 and the retinal ganglion cell (RGC) marker RBPMS (Figure 8B) as well as the horizontal cell marker calbindin (CaBP) (Figure 8C). The signal was apparent in both the nucleus and cytoplasm. These data indicated that the lncRNA Gm35369 was located mainly in RGCs and horizontal cells.

## Discussion

In this study, we analyzed the differential expression patterns of lncRNAs and mRNAs in FDM mouse retinas. The results from RNA FISH localized the target lncRNAs in specific retinal layers and cell types. We hope that this evidence might lay a foundation for future research on myopia. Previously, miRNA profiling of the whole eyes, retinas, and sclerae of mice (Tkatchenko et al., 2016; Mei et al., 2017) and lncRNA-mRNA sequencing of the ocular posterior poles of guinea pigs with experimental myopia have been reported (Geng et al., 2020). Our study focused on the retina rather than the whole eyeball or posterior poles, which reduced the possible heterogeneity from different tissues.

A mouse FDM model was induced for the sequencing analysis in this study, similar to the previously reported experimental myopia model. The Wu’s results showed a myopic shift in the deprived eye with the refractive difference (OD 1.341 ± 0.298 D–OS 6.440 ± 0.292 D) of −5.099 ± 0.239 D (*p* < 0.001), which is slightly smaller than ours (−8.44 ± 4.93 D, *p* < 0.001). In another mice study with shorter deprivation of 10 days, the deprived eyes were induced to myopia of −6.93 ± 2.44 D (*p* < 0.000001) compared to the contralateral control eyes (Tkatchenko et al., 2016); and in a study of 2 weeks deprivation, the refraction difference was about −7 D (Wu et al., 2018). Taken together, these observations suggest a significant myopic shift in the refractive error, even with some variations in each report (possibly due to the animals, measuring method, or equipment), would be induced after monocular visual form deprivation ranging from 10 days to 4 weeks.

In sequencing analysis of eye, a sample size of three replicates or more than three replicates could be justifiable, as the differential gene expression from RNA-seq was successfully validated by qRT-PCT. Moreover, due to the small volume and low RNA content of ocular tissue, some studies pooled samples to get enough RNA for sequencing. For instance, Tkatchenko et al. analyzed the microRNA expression profiling in the retina and sclera of FDM mice by microarray, with the small sample size of only three replicates (3 eyes pooled together per replicate) in parallel (Tkatchenko et al., 2016). Similarly, four sclerae were pooled to form one sample in the bulk transcriptome sequencing (Zhao et al., 2021). Vocale *et al.* identified ligand-gated chloride efflux channels as a major pathway contributing to chick FDM using RNA-seq and gene set enrichment analysis, with four replicates (4 chicks) per condition (Vocale et al., 2021); and sample size of three or four replicates (3 or 4 guinea pig eyes) in the RNA-seq study to investigate the gene expression and pathways in sclera was also reported (Srinivasalu et al., 2018; Zeng et al., 2021). The current study included six replicates per experimental condition (FDM or contralateral control, with 3 retinas pooled per replicate), much more than the previous reported RNA-seq datasets, hoping to increase the confidence of our RNA-seq data. Besides, the validation of expression level of lncRNAs by qRT-PCR and sequencing showed a similar direction between the two techniques.

After the induction of FDM, the refractive error was measured by cycloplegic streak retinoscopy in the current study, which would possibly produce measurement error. Refraction in previous study was assessed by various methods, including automated eccentric infrared photorefractor (Schaeffel et al., 2004; Wu et al., 2018) and streak retinoscopy by optometrist (Huang et al., 2019; Zeng et al., 2021). In our study, the optometrist and the researchers who bled and built the model were different, and optometrist was masked to the treatment status of the eyes during measurements of refractive errors. Moreover, an interocular refractive difference greater than 5 D was considered as successful induction of FDM, which adds to the confidence of the subsequent data. Axial length was measured by SD-OCT (Wu et al., 2015; Wu et al., 2018), MRI (Tkatchenko et al., 2010; Tkatchenko et al., 2013), or even digital caliper and video imaging micrometer in the early days (Barathi et al., 2008). The current study applied OCT to measure the mouse axial length as previous described. Another limitation of the generation of FDM model was that the ocular parameters were only recorded in the end of the deprivation. Future experiments could assess the parameters at different time points (including before the deprivation) during the generation of FDM, to describe the development of myopia in a detailed way. Additionally, the age of initiation of the FDM and its correlation with the outcome of myopia as well as the gene expression profile would be an interesting direction to investigate, since recent study suggest that the mouse retina develops postnatally with dramatic changes from P10 to P120 by using the Claudin5 (a vascular endothelium-specific gene) eGFP transgenic mice and three-dimensional architecture analysis (Rust et al., 2019).

Among the differentially expressed lncRNAs identified (Tables 1, 2), Oip5os1 (OIP5-AS1, transcript ID: ENSMUST00000147425, FC = 115.55), UBXN7 (transcript ID: chr16: 32388756-32392334, FC = 101.58), and Snhg12 (transcript ID: ENSMUST00000128131, FC = 44.14) exhibited significantly increased expression levels, while Junos (transcript ID: XR_003955073.1, FC = 0.016) and another transcript of Snhg12 (transcript ID: ENSMUST00000126380, FC = 0.017) exhibited downregulated expression in FDM retinal tissue samples. The lncRNA OIP5-AS1 is involved in the pathogenesis of a variety of diseases, including colon cancer (Wang Y. et al., 2021), prostate cancer (Zhang Y. et al., 2021), papillary thyroid cancer (Zhang X. et al., 2021), and osteosarcoma (Li et al., 2021), as well as heart failure (Zhuang et al., 2021). More importantly, previous evidence has suggested that OIP5-AS1 might participate in the mechanisms of primary open angle glaucoma (POAG) and cataracts (Zhou et al., 2020) (Jing et al., 2020). Jing *et al* showed that by regulating apoptosis of lens epithelial cells and aggravating lens opacity, OIP5-AS1 can lead to the formation and development of cataracts (Jing et al., 2020). In POAG, OIP5-AS1, as well as three other lncRNAs, have been found to constitute a hub in a lncRNA-miRNA-mRNA competing endogenous RNA (ceRNA) network (Zhou et al., 2020). UBXN7 was upregulated in human epicardial adipose tissue samples from patients with heart failure (Zheng M. L. et al., 2020). Dysregulation of the lncRNA Snhg12 is involved in a variety of pathogeneses, such as those of LDL-induced endothelial cell injury in atherosclerosis (Mao et al., 2021) and endometrial (Cai et al., 2021), gastric (Zhang T. et al., 2021), and hepatocellular cancer (Zhang Q. et al., 2021). Junos is the opposite strand of the proto-oncogene c-Jun, which is the most extensively studied component of AP-1 and plays important roles in cellular physiology, including proliferation, apoptosis, and tumorigenesis (Meng and Xia, 2011). Therefore, the abnormally expressed lncRNAs in FDM retinas might affect the formation and development of myopia.

Moreover, GO analysis of mRNAs revealed that the differentially expressed mRNAs in the FDM retinas were associated with the cellular process, biological regulation, response to stimulus, metabolic process, developmental process, multicellular organismal process, immune system process, localization, locomotion, behavior, rhythmic process, cellular anatomical entity, binding, and catalytic activity terms. Among the top 20 enriched GO terms, the rhythmic process and the structural molecule activity were shown in the down-regulated but not in the up-regulated pattern. Consistent with recent studies, the downregulation of rhythmic processes or circadian processes might play an important role in the disruption of retinal functions and thus lead to myopia formation (Stone et al., 2013). For instance, the circadian rhythmic control of rod-cone electrical coupling switches the receipt of light signals during day and night (Ribelayga et al., 2008), and diurnal rhythms affect eye growth and refractive error development (Chakraborty et al., 2018). Recent ocular studies focusing on circadian clock genes have also identified the close relationship between myopia formation and circadian dysregulation. Retinal-specific knockout of the clock gene *Bmal1* in mice can induce a myopia shift and elongation of the vitreous chamber (Stone et al., 2019). Furthermore, both FDM and LIM in chicks significantly alter the expression of intrinsic circadian clock genes in the retina/RPE/choroid (Stone et al., 2020). In addition, the synchronization of the local circadian rhythm in the retina with the environmental light cycle requires an orphan opsin, OPN5, which has been found to be involved in emmetropization (Jiang et al., 2021). The term of structural molecule activity belongs to the MF (molecular function) and represents the action of a molecule that contributes to the structural integrity of a complex or its assembly within or outside a cell (Mouse Genome Database, www.informatics.jax.org). Annotated terms under the structural molecule activity include extracellular matrix structural constituent (GO:0005201), structural constituent of cytoskeleton (GO:0005200), structural constituent of eye lens (GO:0005212), and relevant terms. Alterations of extracellular matrix (ECM) remodeling and the cytoskeleton constitution in retina and sclera participate in the development of myopia, and it is generally accepted that the vision guided ocular growth *via* a cascade that firstly from chemical signals initiated in the retina and ultimately change the scleral remodeling (Boote et al., 2020). Early genome-wide association study on myopia in Europeans has revealed that LAMA2, a gene involved in the extracellular matrix, associated with the mechanism behind the development of myopia (Kiefer et al., 2013). Recent transcriptomics analysis of retinas from wavelength-induced myopic guinea pigs also suggest that the differentially expressed genes were primarily enriched in the extracellular matrix, and metabolism, receptor activity, and ion binding processes (Wen et al., 2022). It is worthy to investigate the mechanism of extracellular matrix in retina in myopia as well as the visual development by experimental methods. Our GO results also agree with previous evidence from chicks and guinea pigs. Riddell *et al* showed that fatty acid, sphingolipid, citrate, and mitochondrial metabolism pathways were strongly altered (bidirectionally; up- or downregulated) in retina/RPE/choroid samples from chicks with lens-induced myopia (Riddell et al., 2016). In addition, the levels of retinoic acid and retinaldehyde dehydrogenase-2 from retinoid acid metabolism are changed in the retinas of guinea pigs with lens-induced myopia, with the latter especially altered in the outer plexiform layer (Mao et al., 2012). These signaling pathways exhibiting strong differential expression in the process of myopia might serve key functions in myopic retinas and merit further study.

The G-protein coupled receptor (GPCR) family member muscarinic acetylcholine receptor plays roles in mediating the development of myopia. Early evidence from FDM Syrian hamsters suggested that the muscarinic receptor M(3) might play important roles in the pathogenesis of myopia (Lin et al., 2012). A recent study involving experimental myopia with a mammalian model demonstrated that inhibition of myopia by muscarinic antagonists involved mainly M(1) and M(4) muscarinic receptor signaling (Arumugam and McBrien, 2012). However, Carr *et al* showed that muscarinic antagonist-mediated blockade of human α2A-adrenergic receptor signaling seemed to be able to inhibit chick FDM, but antagonists of the M(4) subtype did not (Carr et al., 2018). In addition, α2A-adrenoceptor agonists have been shown to be effective in inhibiting chick FDM, suggesting that adrenergic receptors are involved in myopia and visual processes (Carr et al., 2019). The KEGG enrichment pathway analysis showed that the interacting genes were enriched in chemokine signaling, GPCRs, intrinsic component of membrane, sensory perception, and catalytic activity-related pathways (Figure 5), indicating the intrinsic and complicated roles of GPCRs in myopia.

Coexpression analysis of the four qRT-PCR-validated lncRNAs showed that XR_377255.2 was correlated with Vcan (Versican), a critical extracellular matrix regulator of immunity and inflammation (Wight et al., 2020). Accumulating studies have suggested the important role of Vcan in cancer growth and metastasis in cancers such as ovarian, breast, and pancreatic cancers (Salem et al., 2018; Zhang et al., 2019; Gao et al., 2020). Although the samples in this study were retinas and previous reports of extracellular matrix-related genes, such as matrix metallopeptidase-2 and inhibitor of metalloproteinase 2, were from sclerae (Geng et al., 2020), it can be speculated that there might be a close relationship between the alteration of Vcan in the retina and the abnormal regulation of the scleral extracellular matrix. The coexpression analysis also revealed that the lncRNA XR_003955073.1 was correlated with downregulation of the mRNA TREM2, triggering receptor expressed in myeloid cells/microglia-2, which is a transmembrane-spanning sensor receptor critical for Aβ42-peptide clearance. In a study by Bhattacharjee *et al.*, TREM2 deficits in the retina and in oxidatively stressed microglia promoted the pathogenesis of amyloidogenesis in age-related macular degeneration (AMD) (Bhattacharjee et al., 2016). However, the pathogenic roles of TREM2 in myopic retinas remain unknown. In addition, CMIP, C-Maf-inducing protein, was correlated with downregulation of the lncRNA XR_866459.4 in the context of myopia. A previous study has suggested that CMIP is expressed in the nervous system and interacts with NF-κB, which is dysregulated in myopia (Lin et al., 2016; Ollero and Sahali, 2021).

Noncoding RNAs can play essential regulatory roles in many biological processes by acting as competing endogenous RNAs (ceRNAs) to suppress miRNAs by preventing them from interacting with target mRNAs (Grull and Masse, 2019). Several ceRNA pairs have been discovered and studied in the contexts of ocular diseases or pathogenesis. A previous report has shown that the lncRNA-MALAT1/miRNA-204-5p ceRNA mechanism is involved in the regulation of epithelial-mesenchymal transition of lens epithelial cells (Peng et al., 2021). The lncRNA MIR7-3HG can modulate miR-27a-3p/PEG10 and promote retinoblastoma progression (Ding et al., 2020). An integrative analysis of the lncRNA ceRNA network in human trabecular meshwork cells under oxidative stress revealed that 70 lncRNAs and 558 mRNAs were significantly dysregulated in HTMCs under oxidative stress compared to the control conditions (Yao et al., 2020). Moreover, ceRNA crosstalk between the lncRNA TUG1 and miRNA-145 has been found to be involved in the suppression of retinal microvascular endothelial cells under high-glucose conditions (Shi et al., 2021). In profiling retinal lncRNAs during myopia progression, we found dysregulation of lncRNAs and their cellular localization, which lays a foundation for further study of possible ceRNA crosstalk in the myopic eye.

Prior studies have suggested that the neurons of the inner retina play an important role in that process (Chen et al., 2006). RGCs and horizontal cells have been studied under the conditions of emmetropization and myopia progression in animal models. Altered cell-cell coupling by the gap junction protein connexin 36 in horizontal cells in the inner plexiform layer (IPL) has been found to play important roles in emmetropization and FDM in guinea pigs, as the uncoupling agent 18-β-GA induces myopic shifts and FDM decreases total connexin 36 levels and phosphorylation (Zhi et al., 2021). In addition, a recent study showed that stimulation of RGCs expressing neuropsin (OPN5) with violet light prevented experimental myopia in mice (Jiang et al., 2021). We found here that the downregulated lncRNA Gm35369 was located mainly in RGCs and horizontal cells. These results indicate that dysregulation of lncRNAs in specific cellular backgrounds is involved in myopia progression. However, the role of Gm35369 in these cell types remains unclear. Further knockdown or overexpression studies with RGCs or horizontal cell lines would help elucidate the mechanisms.

As with all transcriptomic profiling analyses, there were limitations to the present study. First, among the large numbers of differentially expressed lncRNAs and mRNAs, only 4 lncRNAs were verified by qRT–PCR, while the expression levels of the others remained uncertain. The predicted correlated mRNAs were not verified experimentally, so it is worth testing the levels of these mRNAs in the future. Second, some lncRNA levels verified by qRT-PCR were not consistent with the RNA sequencing data. For instance, the fold change (FC = FDM/ctrl) of the lncRNA XR_866459.4 in qRT-PCR was 0.45 (suggesting decreased expression in the myopic retina), while the FC from the sequencing data was 10.78 (data not shown). Thus, the RNA sequencing results can only be regarded as a reference dataset, and further experiments are needed to confirm the lncRNA targets of interest. Second, although the lncRNA alterations dynamically changed over the course of visual development, we tested the changes in lncRNA levels at only one time point (after FDM induction for 4 weeks). Third, *in vitro* analysis of specific cultured retinal cell lines is required to identify specific pathways and targets for possible gene-based approaches or drugs to modulate the pathogenesis of myopia. Finally, it is necessary to identify the levels of these lncRNAs in patients with myopia.

## Conclusion

Overall, this study analyzed the aberrant expression profiles of lncRNAs and mRNAs in the retinas of FDM mouse models with high-throughput sequencing. The potential roles of the significantly differentially expressed lncRNAs might be related to sensory perception of chemical stimuli, the G-protein coupled receptor signaling pathway, cytokine-cytokine receptor interactions, retinol metabolism, olfactory transduction, metabolism of xenobiotics by cytochrome P450, chemical carcinogenesis, tyrosine metabolism, and the proteasome, which might contribute to retinal myopic pathogenesis. We have preliminarily shown that the lncRNA Gm35369 is mainly located in RGCs and horizontal cells. These findings expand our understanding of lncRNAs in the myopic retina. By revealing a number of candidate target genes and the localization of lncRNAs in specific cell types, this study provides valuable evidence and will support future *in vitro/vivo* studies to investigate the potential mechanisms in myopia.

## Data Availability

The dataset presented in this study can be found in online repository. The name of the repository and accession number can be found below: NCBI SRA BioProject, PRJNA832969 (released upon publication).
