# Supplementary figures and images for: RNA-sequencing analysis reveals the long noncoding RNA profile in the mouse myopic retina
Source: Front Genet. 2022 Oct 13;13:1014031. doi: 10.3389/fgene.2022.1014031 (PMC9606684; doi:10.3389/fgene.2022.1014031)

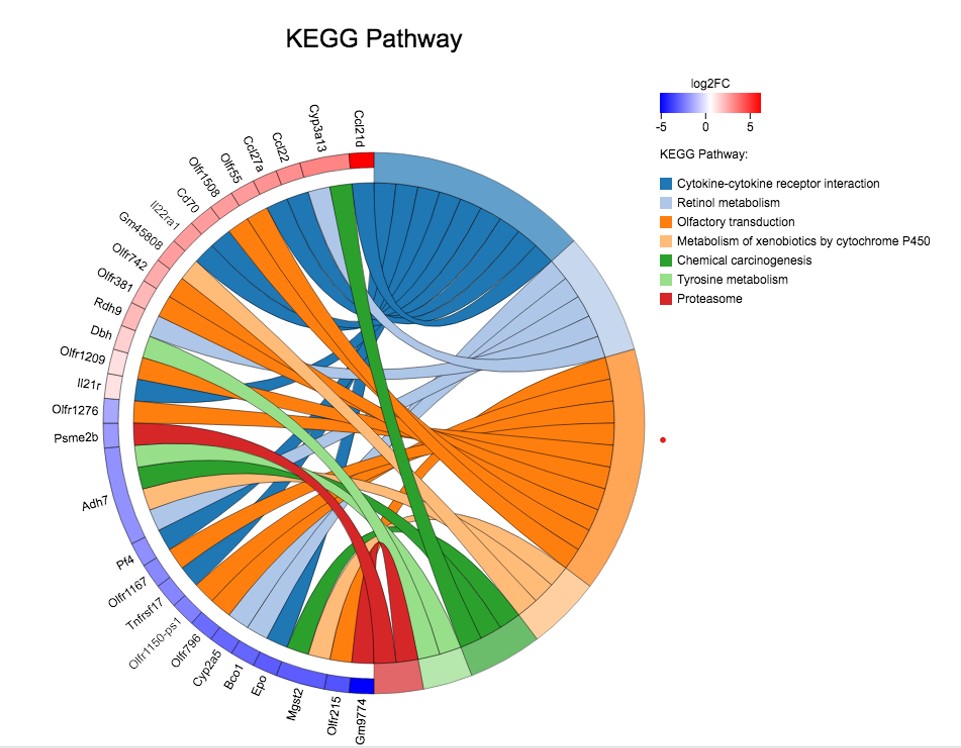

Supplement: Supplementary file 2 [file Image3.JPEG]

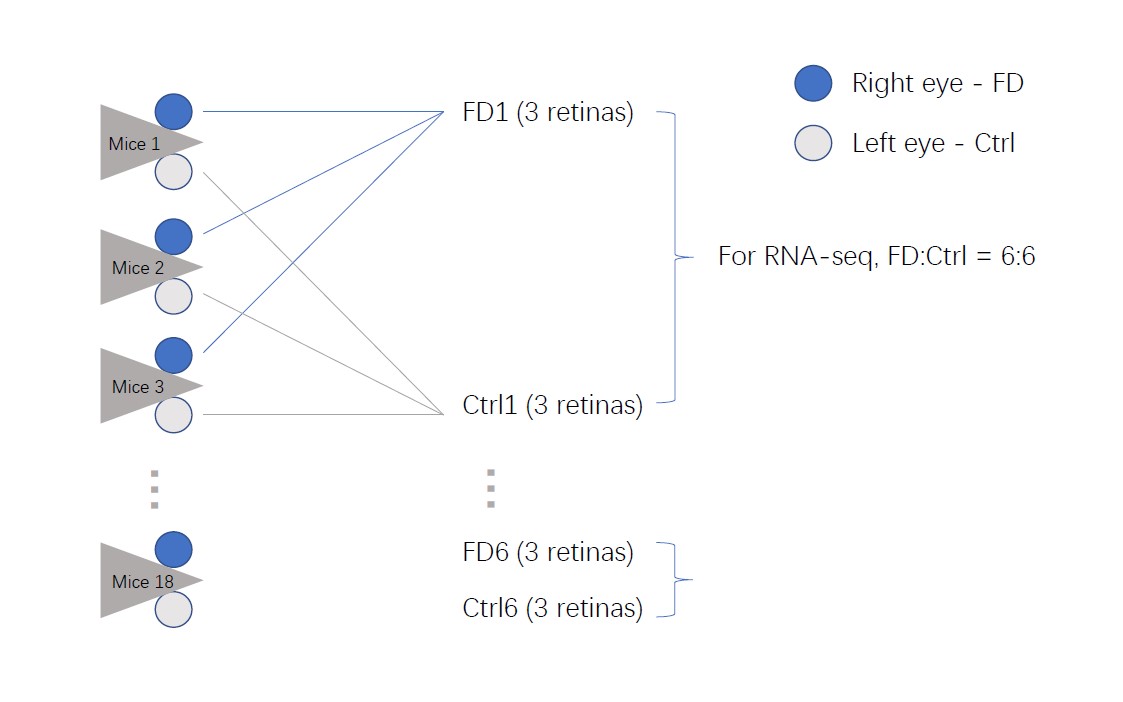

Supplement: Supplementary file 5 [file Image1.JPEG]

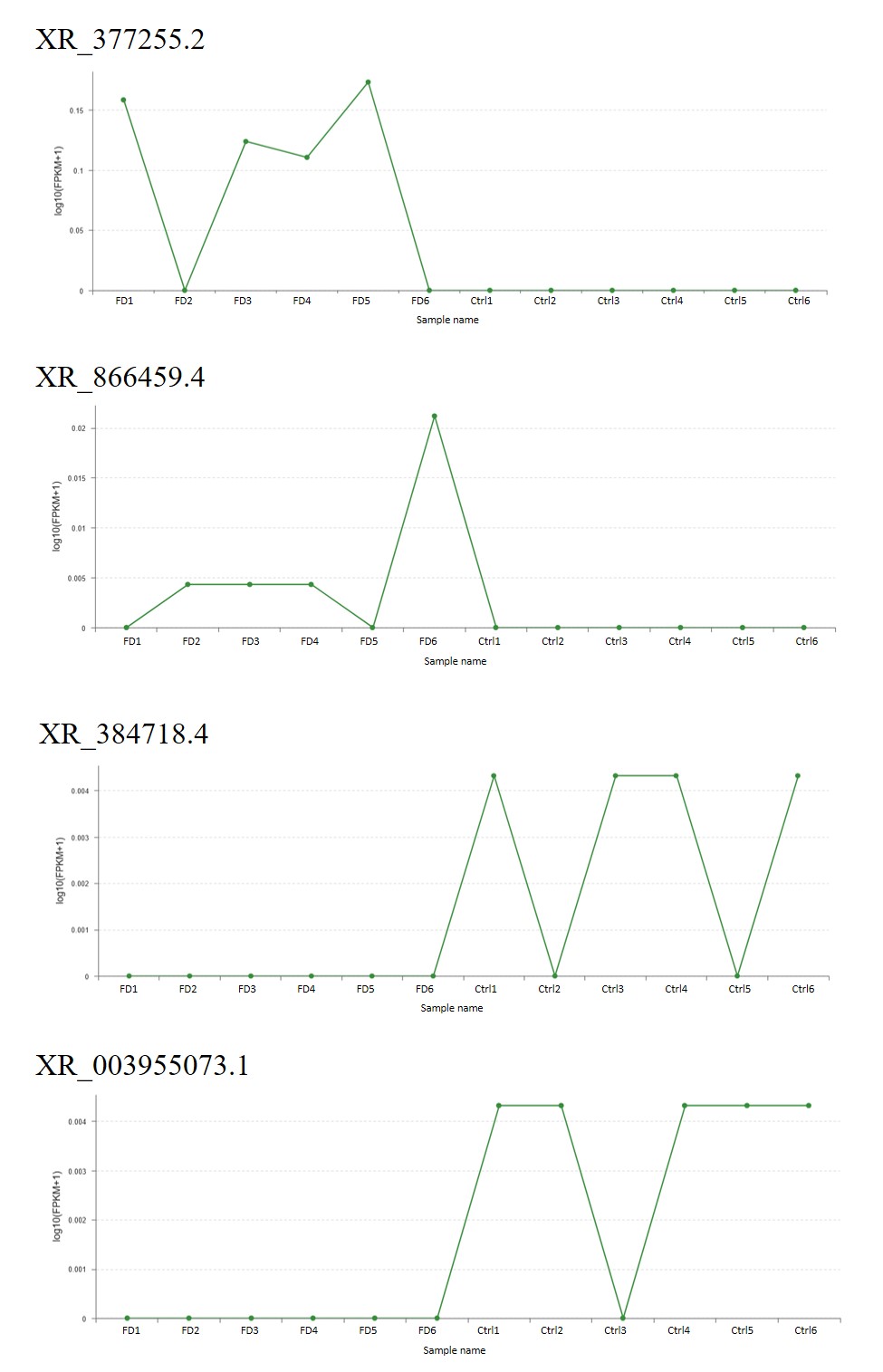

Supplement: Supplementary file 6 [file Image4.JPEG]

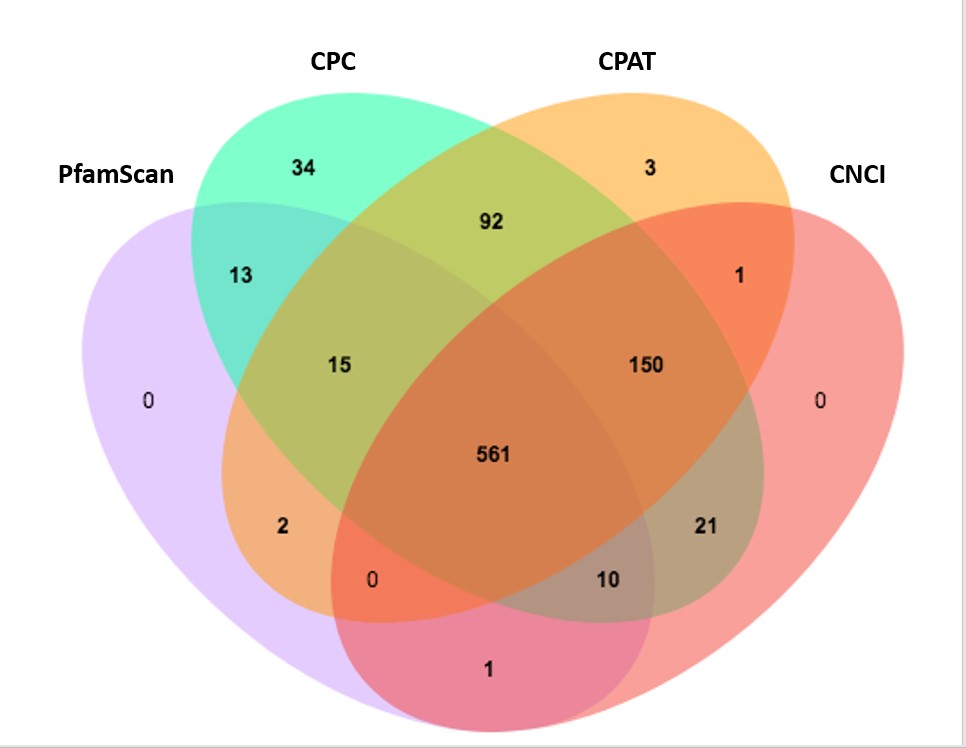

Supplement: Supplementary file 7 [file Image2.JPEG]
