# Supplementary material for: RNA-sequencing analysis reveals the long noncoding RNA profile in the mouse myopic retina
Source: Front Genet. 2022 Oct 13;13:1014031. doi: 10.3389/fgene.2022.1014031 (PMC9606684; doi:10.3389/fgene.2022.1014031)
Supplement: Supplementary file 4 [file Table1.DOCX]

| **Gene symbol** | **Transcript ID** | **Forward primer, 5’-3’** | **Reverse Primer, 5’-3’** | **Product length (bp)** |
| --- | --- | --- | --- | --- |
| Gm15411 | XR_377255.2 | GTGCAGAGGACCCTTCTTTGTC | ATGCTCCAGTGACTCAGTCGC | 83 |
| Gm39857 | XR_866459.4 | TGCGAGGCATGGATGTAATC | GTGTCTAGGACGGACTGCTC | 59 |
| Junos | XR_003955073.1 | AGAACTTGGGTAAGCCGCC | CAGAGCACTGTTCTTGGGCA | 236 |
| Gm35369 | XR_384718.4 | TGTAGCAGCAACTAAGTGGAAG | TCCTAAGAGTCGGGTCTTCTT | 61 |
| GAPDH |  | GGTTGTCTCCTGCGACTTCA | TGGTCCAGGGTTTCTTACTCC | 183 |
| Gm35369  (for RNA-FISH) | XR_384718.4 | F: 5’-TGCAGTCATGGCCTTGATCCTTCCACTTAGTTGCT  (ttt AATACTCTC AATACTCTC) _30_-3’ | |  |
|  |  | R: 5’-DIG-ttGAGAGTATTGAGAGTATT-3’ with double-labeled | |  |

**Supplemental Table 1**

Primer sequences for qRT-PCR or FISH analysis.
